# *N*-{1-[(3-Bromo­prop­yl)amino­carbon­yl]eth­yl}-2-(2-nitro­benzene­sulfonamido)propionamide

**DOI:** 10.1107/S1600536809034291

**Published:** 2009-09-05

**Authors:** Ravula Thirupathi, Damodara N. Reddy, S. Brinda, Erode N. Prabhakaran

**Affiliations:** a Department of Organic Chemistry, Indian Institute of Science, Bangalore 560 012, Karnataka, India; b Solid State and Structural Chemistry Unit, Indian Institute of Science, Bangalore 560 012, Karnataka, India

## Abstract

In the title compound, C_15_H_21_BrN_4_O_6_S, all three NH groups are involved in inter­molecular N—H⋯O inter­actions which, together with two inter­molecular C—H⋯O contacts, lead to a continuous anti­parallel β-sheet structure. There are no π–π inter­actions between mol­ecules, and two C—H⋯π inter­actions primarily govern the linkage between sheets.

## Related literature

For conformationally restricted peptide analogues, see: Belvisi *et al.* (2000 ▶); Ripka *et al.* (1993 ▶). For C-H⋯π inter­actions in crystals and peptides, see: Ciunik *et al.* (1998 ▶); Görbitz (1989 ▶); Nishio (2004 ▶); Nishio & Hirota (1989 ▶). For the correlation between peptide sequences and folds, see: Venkatraman *et al.* (2001 ▶); Wilmot & Thornton (1988 ▶). For bond angles in β-strand structures, see: Loughlin *et al.* (2004 ▶).
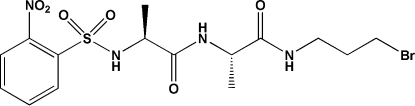


## Experimental

### 

#### Crystal data

C_15_H_21_BrN_4_O_6_S
*M* *_r_* = 465.33
Orthorhombic, 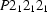

*a* = 9.4467 (4) Å
*b* = 12.7438 (5) Å
*c* = 17.3257 (7) Å
*V* = 2085.79 (15) Å^3^
*Z* = 4
Mo *K*α radiation
μ = 2.11 mm^−1^
*T* = 292 K
0.30 × 0.20 × 0.10 mm

#### Data collection

Bruker SMART CCD area-detector diffractometer
Absorption correction: multi-scan (*SADABS*; Sheldrick, 1997 ▶) *T*_min_ = 0.571, *T*_max_ = 0.817
33853 measured reflections
4107 independent reflections
3007 reflections with *I* > 2σ(*I*)
*R*_int_ = 0.054

#### Refinement

*R*[*F* ^2^ > 2σ(*F* ^2^)] = 0.047
*wR*(*F* ^2^) = 0.138
*S* = 1.10
4107 reflections
247 parameters
H-atom parameters constrained
Δρ_max_ = 0.50 e Å^−3^
Δρ_min_ = −0.51 e Å^−3^
Absolute structure: Flack (1983 ▶), 1763 Friedel pairs
Flack parameter: −0.011 (13)

### 

Data collection: *SMART* (Bruker, 2004 ▶); cell refinement: *SAINT* (Bruker, 2004 ▶); data reduction: *SAINT*; program(s) used to solve structure: *SHELXS97* (Sheldrick, 2008 ▶); program(s) used to refine structure: *SHELXL97* (Sheldrick, 2008 ▶); molecular graphics: *ORTEP-3 for Windows* (Farrugia, 1999 ▶) and *CAMERON* (Watkin *et al.*, 1993 ▶); software used to prepare material for publication: *PLATON* (Spek, 2009 ▶).

## Supplementary Material

Crystal structure: contains datablocks global, I. DOI: 10.1107/S1600536809034291/bg2284sup1.cif

Structure factors: contains datablocks I. DOI: 10.1107/S1600536809034291/bg2284Isup2.hkl

Additional supplementary materials:  crystallographic information; 3D view; checkCIF report

## Figures and Tables

**Table 1:** Hydrogen-bond geometry (Å, °)

*D*—H⋯*A*	*D*—H	H⋯*A*	*D*⋯*A*	*D*—H⋯*A*
N2—H2*A*⋯O6^i^	0.86	2.07	2.884 (4)	158
N3—H3*A*⋯O6	0.86	2.54	2.829 (4)	100
N3—H3*A*⋯O5^ii^	0.86	2.07	2.899 (4)	162
N4—H4*A*⋯O4^i^	0.86	2.35	3.165 (5)	159
C2—H2⋯O4	0.93	2.49	2.867 (6)	104
C7—H7⋯O5^ii^	0.98	2.34	3.193 (5)	145
C10—H10⋯O4^i^	0.98	2.51	3.431 (5)	156
C13—H13*A*⋯O6	0.97	2.46	2.802 (6)	100
C13—H13*B*⋯O3^iii^	0.97	2.60	3.496 (6)	154
C11—H11*A*⋯*Cg*	3.39	0.96	3.922 (6)	117
C11—H11*B*⋯*Cg*^i^	3.27	0.96	3.857 (6)	121

Symmetry codes: (i) 
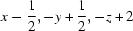
; (ii) 
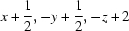
; (iii) 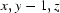. *Cg* is the centroid of the C1–C6 ring.

**Fig. 1.**
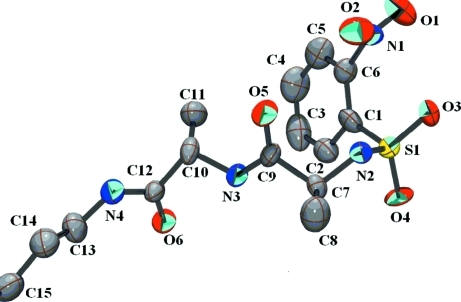
View of the title compound with the atom numbering scheme. Displacement ellipsoids for non-H atoms are drawn at the 50% probability level. H atoms have been omitted for clarity.

**Fig. 2.**
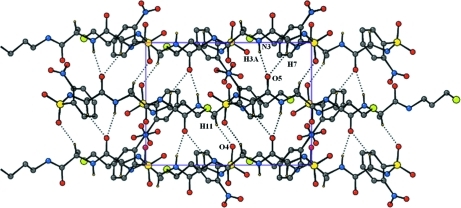
Packing diagram of (I). The dotted lines indicate intermolecular C—H···O and N—H···O interactions.

## supplementary crystallographic information

### Comment

The title compound is a precursor for making conformationally restricted dipeptide analogues, which are essential for many molecular recognition events including interactions between antigens and antibodies, peptide hormones and their receptors, and enzymes and their corresponding substrates (Ripka *et al.*, 1993; Belvisi *et al.*, 2000). The dipeptide sequence Ala-Ala has a low frequency of appearance in the conformationally ordered regions of polypeptides (Wilmot & Thornton, 1988; Venkatraman *et al.*, 2001). The sulfonamide group is known to render conformational ordering in peptides and many sulfonamides are crystalline in nature. The title compound was synthesized to investigate the ordering rendered to Ala-Ala dipeptide by the N-nosyl (2-nitro-benzenesulfonylamino) protecting group. In the crystal structure all the three NH groups of the molecule are involved in intermolecular N—H···O interactions.

The two adjacent amide N-H bonds, N_3_—H_3_ and N_4_—H_4_, that flank the C- terminal alanine in the title compound are antiperiplanar to each other. The phi, psi angles for the C-terminal alanine are phi = -151.9 (5)°, psi = 130.4 (2)°. These angles and the H3-N3-N4-H4 dihedral angle (166.1 (3)°) are within the limits of those found in b-strand structures (Loughlin *et al.*, 2004). On the other hand, the two adjacent N-H bonds N_2_—H_2_ and N_3_—H_3_ that flank the N-terminal alanine are slightly distorted away from ideal antiperiplanarity (H2-N2-N3-H3 dihedral angle = 150.2 (5)°). The phi, psi angles for the N-terminal alanine are phi = 95.6 (2)°, psi = 137.8 (7)°. The distortion from the ideal phi value for a beta-strand near N2 is probably due to the fact that N2 is bonded to a sulfonyl group rather than an acyl group.

The strands are arranged in a head-to-tail fashion, with three intermolecular N—H···O interactions and two intermolecular C—H···O interactions (Table 1). These interactions are between adjacent strands and assist in forming a continuous beta-sheet structure. The C1—S1—N2—C7 torsion angle is 62.9 (3)°. This orients the phenyl ring at a dihedral angle of 73.9 (1)° from the mean plane of the rest of the molecule. The crystal structure is stabilized by two C—H···π interactions. One is intermolecular (C_11_—C_g_ = 3.85 A°, Cg: the centroid of the phenyl ring) and the other is intramolecular (C_11_—C_g_ = 3.92 A°). There are no π–π interactions between the phenyl rings and the interactions between the sheets are solely governed by the C—H···π interactions.

### Experimental

To a stirring solution of 2-[2'-(2-nitrosulfonylamido)-propionamido]-propanoic acid (650 mg, 1.88 mmol) in THF (10 ml) at 258 K was added *N*-methyl morpholene (0.31 ml, 2.82 mmol) followed by ethylchloroformate (0.18 ml,1.93 mmol) under N_2_ atmosphere. After two minutes a solution of 3-bromopropan-1-ammonium bromide (536 mg, 2.44 mmol) and *N*-Methyl morpholene (0.51 ml, 4.7 mmol) in a mixture of DMF/THF (1.5/3 ml) were added to the mixture and stirred for 10 min. The reaction mixture was warmed to room temperature and stirred for further 8 h. THF was removed under reduced pressure and the resulting residue was diluted with EtOAc (10 ml) and washed with saturated aqueous citric acid solution (5 ml), saturated aqueous NaHCO_3_ (5 ml) solution and dried (anhydrous Na_2_SO_4_). The solvent was removed under reduced pressure and the resulting residue was purified by silica gel flash column chromatography (EtOAc/Hexane:1/2) to obtain the title compound as a colorless solid 392 mg (0.84 mmol, 45%) (m.p. 404 K). Needle like crystals were obtained for the isolated compound by slow evaporation at room temperature from a solution in 2-propanol (2.1 m*M*).

### Refinement

All the H atoms were positioned geometrically with C—H bond lengths of 0.93 (3)–0.97 (3) Å, and refined using a riding model approximation with *U*_iso_(H) = 1.2 *U*_eq_(C) or 1.5 *U*_eq_(C) for methyl H atoms.

### Crystal data

**Table:**

C_15_H_21_BrN_4_O_6_S	*D*_x_ = 1.479 Mg m^−^^3^
*M**_r_* = 465.33	Melting point: 404 K
Orthorhombic, *P*2_1_2_1_2_1_	Mo *K*α radiation, λ = 0.71073 Å
Hall symbol: P 2ac 2ab	Cell parameters from 3007 reflections
*a* = 9.4467 (4) Å	θ = 2.0–26.0°
*b* = 12.7438 (5) Å	µ = 2.11 mm^−^^1^
*c* = 17.3257 (7) Å	*T* = 292 K
*V* = 2085.79 (15) Å^3^	Needle, colourless
*Z* = 4	0.30 × 0.20 × 0.10 mm
*F*(000) = 948	

### Data collection

**Table:**

Bruker SMART CCD area-detector diffractometer	4107 independent reflections
Radiation source: fine-focus sealed tube	3007 reflections with *I* > 2σ(*I*)
graphite	*R*_int_ = 0.054
φ and ω scans	θ_max_ = 26.0°, θ_min_ = 2.0°
Absorption correction: multi-scan (*SADABS*; Sheldrick, 1997)	*h* = −11→11
*T*_min_ = 0.571, *T*_max_ = 0.817	*k* = −15→15
33853 measured reflections	*l* = −21→21

### Refinement

**Table:**

Refinement on *F*^2^	Secondary atom site location: difference Fourier map
Least-squares matrix: full	Hydrogen site location: inferred from neighbouring sites
*R*[*F*^2^ > 2σ(*F*^2^)] = 0.047	H-atom parameters constrained
*wR*(*F*^2^) = 0.138	*w* = 1/[σ^2^(*F*_o_^2^) + (0.08*P*)^2^] where *P* = (*F*_o_^2^ + 2*F*_c_^2^)/3
*S* = 1.10	(Δ/σ)_max_ < 0.001
4107 reflections	Δρ_max_ = 0.50 e Å^−^^3^
247 parameters	Δρ_min_ = −0.51 e Å^−^^3^
0 restraints	Absolute structure: Flack (1983), 1763 Friedel pairs
Primary atom site location: structure-invariant direct methods	Flack parameter: −0.011 (13)

### Special details

**Table:**

Geometry. All e.s.d.'s (except the e.s.d. in the dihedral angle between two l.s. planes) are estimated using the full covariance matrix. The cell e.s.d.'s are taken into account individually in the estimation of e.s.d.'s in distances, angles and torsion angles; correlations between e.s.d.'s in cell parameters are only used when they are defined by crystal symmetry. An approximate (isotropic) treatment of cell e.s.d.'s is used for estimating e.s.d.'s involving l.s. planes.
Refinement. Refinement of *F*^2^ against ALL reflections. The weighted *R*-factor *wR* and goodness of fit *S* are based on *F*^2^, conventional *R*-factors *R* are based on *F*, with *F* set to zero for negative *F*^2^. The threshold expression of *F*^2^ > σ(*F*^2^) is used only for calculating *R*-factors(gt) *etc*. and is not relevant to the choice of reflections for refinement. *R*-factors based on *F*^2^ are statistically about twice as large as those based on *F*, and *R*- factors based on ALL data will be even larger.

### Fractional atomic coordinates and isotropic or equivalent isotropic displacement parameters (Å^2^)

**Table:**

	*x*	*y*	*z*	*U*_iso_*/*U*_eq_	
Br1	0.53403 (7)	−0.37246 (5)	1.22175 (4)	0.0837 (3)	
S1	0.50247 (10)	0.51539 (8)	0.93158 (6)	0.0381 (3)	
C1	0.4914 (5)	0.4329 (3)	0.8477 (2)	0.0399 (9)	
C4	0.4882 (8)	0.3109 (5)	0.7161 (3)	0.0774 (18)	
H4	0.4882	0.2697	0.6719	0.093*	
C2	0.6041 (5)	0.3690 (4)	0.8313 (3)	0.0553 (12)	
H2	0.6826	0.3681	0.8637	0.066*	
C6	0.3764 (5)	0.4355 (4)	0.7986 (3)	0.0436 (11)	
C5	0.3759 (6)	0.3765 (4)	0.7309 (3)	0.0633 (14)	
H5	0.3009	0.3815	0.6963	0.076*	
C3	0.6009 (6)	0.3060 (4)	0.7667 (4)	0.0692 (16)	
H3	0.6751	0.2599	0.7571	0.083*	
O3	0.4231 (3)	0.6086 (2)	0.91578 (19)	0.0506 (8)	
O4	0.6505 (3)	0.5244 (3)	0.9481 (2)	0.0550 (9)	
O1	0.1799 (4)	0.4744 (3)	0.8693 (2)	0.0690 (10)	
O2	0.2243 (5)	0.5726 (3)	0.7713 (3)	0.0828 (12)	
N1	0.2509 (4)	0.5005 (4)	0.8145 (3)	0.0526 (10)	
N2	0.4295 (3)	0.4562 (2)	1.0023 (2)	0.0345 (8)	
H2A	0.3557	0.4835	1.0233	0.041*	
C9	0.4065 (4)	0.2648 (3)	0.9969 (3)	0.0363 (9)	
C7	0.4847 (4)	0.3563 (3)	1.0325 (2)	0.0335 (8)	
H7	0.5856	0.3507	1.0199	0.040*	
C8	0.4666 (7)	0.3526 (4)	1.1197 (3)	0.0709 (16)	
H8A	0.5211	0.4078	1.1430	0.106*	
H8B	0.4988	0.2860	1.1388	0.106*	
H8C	0.3685	0.3617	1.1325	0.106*	
O5	0.2778 (3)	0.2645 (3)	0.9909 (2)	0.0607 (10)	
N3	0.4858 (3)	0.1824 (2)	0.9757 (2)	0.0362 (8)	
H3A	0.5765	0.1867	0.9789	0.043*	
C12	0.5198 (4)	−0.0043 (3)	0.9661 (2)	0.0383 (9)	
C10	0.4212 (4)	0.0847 (3)	0.9472 (3)	0.0432 (11)	
H10	0.3307	0.0735	0.9735	0.052*	
C11	0.3953 (6)	0.0907 (4)	0.8584 (3)	0.0611 (14)	
H11A	0.3342	0.1489	0.8471	0.092*	
H11B	0.3518	0.0268	0.8411	0.092*	
H11C	0.4841	0.1001	0.8323	0.092*	
O6	0.6452 (3)	−0.0016 (2)	0.94723 (19)	0.0473 (8)	
N4	0.4607 (4)	−0.0872 (3)	1.0009 (2)	0.0497 (9)	
H4A	0.3710	−0.0865	1.0092	0.060*	
C13	0.5414 (6)	−0.1783 (4)	1.0252 (3)	0.0582 (13)	
H13A	0.6400	−0.1582	1.0307	0.070*	
H13B	0.5362	−0.2312	0.9851	0.070*	
C14	0.4926 (6)	−0.2250 (4)	1.0989 (3)	0.0663 (14)	
H14A	0.3973	−0.2521	1.0924	0.080*	
H14B	0.4901	−0.1712	1.1385	0.080*	
C15	0.5908 (7)	−0.3138 (5)	1.1244 (3)	0.0698 (15)	
H15A	0.6866	−0.2872	1.1287	0.084*	
H15B	0.5905	−0.3686	1.0855	0.084*	

### Atomic displacement parameters (Å^2^)

**Table:**

	*U*^11^	*U*^22^	*U*^33^	*U*^12^	*U*^13^	*U*^23^
Br1	0.0935 (5)	0.0790 (5)	0.0787 (4)	−0.0189 (3)	−0.0056 (3)	0.0102 (4)
S1	0.0347 (5)	0.0326 (5)	0.0469 (6)	−0.0058 (4)	−0.0054 (4)	0.0037 (4)
C1	0.042 (2)	0.037 (2)	0.041 (2)	0.0002 (19)	0.0050 (19)	0.0055 (18)
C4	0.111 (5)	0.065 (3)	0.056 (3)	−0.004 (4)	0.031 (4)	−0.018 (3)
C2	0.049 (3)	0.053 (3)	0.064 (3)	0.003 (2)	0.013 (2)	0.002 (3)
C6	0.048 (3)	0.043 (3)	0.039 (3)	−0.006 (2)	0.0000 (18)	−0.004 (2)
C5	0.075 (4)	0.066 (3)	0.049 (3)	−0.011 (3)	0.003 (2)	−0.003 (3)
C3	0.071 (3)	0.054 (3)	0.083 (4)	0.011 (3)	0.026 (3)	−0.006 (3)
O3	0.060 (2)	0.0286 (15)	0.063 (2)	0.0015 (14)	−0.0122 (15)	0.0029 (15)
O4	0.0340 (15)	0.062 (2)	0.069 (2)	−0.0152 (15)	−0.0057 (14)	0.0093 (18)
O1	0.058 (2)	0.087 (3)	0.062 (2)	0.013 (2)	0.0060 (19)	−0.007 (2)
O2	0.097 (3)	0.073 (3)	0.078 (3)	0.020 (2)	−0.024 (2)	0.021 (3)
N1	0.052 (2)	0.059 (3)	0.047 (2)	0.0065 (19)	−0.0159 (19)	−0.008 (2)
N2	0.0293 (17)	0.0308 (17)	0.043 (2)	−0.0017 (13)	0.0021 (14)	−0.0028 (15)
C9	0.029 (2)	0.030 (2)	0.050 (3)	−0.0036 (16)	0.0025 (18)	0.0012 (19)
C7	0.0304 (19)	0.0297 (19)	0.040 (2)	0.0039 (16)	−0.0006 (17)	−0.0001 (17)
C8	0.106 (5)	0.059 (3)	0.047 (3)	0.002 (3)	−0.004 (3)	0.000 (2)
O5	0.0262 (15)	0.0428 (19)	0.113 (3)	0.0017 (13)	0.0000 (17)	−0.021 (2)
N3	0.0216 (15)	0.0293 (16)	0.058 (2)	−0.0037 (14)	−0.0016 (15)	−0.0067 (15)
C12	0.034 (2)	0.032 (2)	0.050 (2)	−0.0031 (17)	−0.0015 (17)	−0.0110 (18)
C10	0.030 (2)	0.029 (2)	0.070 (3)	−0.0064 (16)	0.003 (2)	−0.006 (2)
C11	0.075 (4)	0.049 (3)	0.059 (3)	0.000 (2)	−0.023 (3)	−0.011 (3)
O6	0.0345 (15)	0.0413 (17)	0.066 (2)	−0.0021 (13)	0.0056 (13)	−0.0032 (16)
N4	0.041 (2)	0.040 (2)	0.068 (2)	−0.0050 (16)	0.0005 (17)	0.0037 (18)
C13	0.063 (3)	0.038 (2)	0.074 (4)	0.001 (2)	0.003 (3)	−0.001 (2)
C14	0.062 (3)	0.052 (3)	0.086 (4)	−0.009 (3)	0.001 (3)	0.001 (3)
C15	0.089 (4)	0.068 (4)	0.053 (3)	−0.002 (3)	−0.004 (3)	−0.002 (3)

### Geometric parameters (Å, °)

**Table:**

Br1—C15	1.921 (6)	C7—H7	0.9800
S1—O3	1.431 (3)	C8—H8A	0.9600
S1—O4	1.431 (3)	C8—H8B	0.9600
S1—N2	1.595 (3)	C8—H8C	0.9600
S1—C1	1.797 (4)	N3—C10	1.471 (5)
C1—C2	1.370 (7)	N3—H3A	0.8600
C1—C6	1.380 (6)	C12—O6	1.229 (5)
C4—C5	1.375 (8)	C12—N4	1.338 (5)
C4—C3	1.381 (9)	C12—C10	1.503 (6)
C4—H4	0.9300	C10—C11	1.560 (6)
C2—C3	1.377 (7)	C10—H10	0.9800
C2—H2	0.9300	C11—H11A	0.9600
C6—C5	1.393 (7)	C11—H11B	0.9600
C6—N1	1.473 (6)	C11—H11C	0.9600
C5—H5	0.9300	N4—C13	1.452 (6)
C3—H3	0.9300	N4—H4A	0.8600
O1—N1	1.209 (6)	C13—C14	1.482 (8)
O2—N1	1.211 (6)	C13—H13A	0.9700
N2—C7	1.472 (5)	C13—H13B	0.9700
N2—H2A	0.8600	C14—C15	1.529 (8)
C9—O5	1.220 (5)	C14—H14A	0.9700
C9—N3	1.342 (5)	C14—H14B	0.9700
C9—C7	1.513 (5)	C15—H15A	0.9700
C7—C8	1.521 (6)	C15—H15B	0.9700
			
O3—S1—O4	118.90 (19)	C7—C8—H8C	109.5
O3—S1—N2	108.22 (19)	H8A—C8—H8C	109.5
O4—S1—N2	107.87 (19)	H8B—C8—H8C	109.5
O3—S1—C1	107.47 (19)	C9—N3—C10	121.5 (3)
O4—S1—C1	105.4 (2)	C9—N3—H3A	119.3
N2—S1—C1	108.64 (18)	C10—N3—H3A	119.3
C2—C1—C6	119.8 (4)	O6—C12—N4	122.9 (4)
C2—C1—S1	118.1 (4)	O6—C12—C10	121.2 (4)
C6—C1—S1	122.0 (3)	N4—C12—C10	115.8 (3)
C5—C4—C3	120.3 (5)	N3—C10—C12	108.0 (3)
C5—C4—H4	119.8	N3—C10—C11	110.8 (4)
C3—C4—H4	119.8	C12—C10—C11	110.4 (4)
C1—C2—C3	119.9 (5)	N3—C10—H10	109.2
C1—C2—H2	120.1	C12—C10—H10	109.2
C3—C2—H2	120.1	C11—C10—H10	109.2
C1—C6—C5	120.6 (5)	C10—C11—H11A	109.5
C1—C6—N1	122.1 (4)	C10—C11—H11B	109.5
C5—C6—N1	117.3 (4)	H11A—C11—H11B	109.5
C4—C5—C6	118.8 (5)	C10—C11—H11C	109.5
C4—C5—H5	120.6	H11A—C11—H11C	109.5
C6—C5—H5	120.6	H11B—C11—H11C	109.5
C2—C3—C4	120.4 (5)	C12—N4—C13	122.9 (4)
C2—C3—H3	119.8	C12—N4—H4A	118.6
C4—C3—H3	119.8	C13—N4—H4A	118.6
O1—N1—O2	125.4 (5)	N4—C13—C14	114.1 (5)
O1—N1—C6	116.0 (4)	N4—C13—H13A	108.7
O2—N1—C6	118.6 (4)	C14—C13—H13A	108.7
C7—N2—S1	122.0 (3)	N4—C13—H13B	108.7
C7—N2—H2A	119.0	C14—C13—H13B	108.7
S1—N2—H2A	119.0	H13A—C13—H13B	107.6
O5—C9—N3	122.1 (4)	C13—C14—C15	111.0 (5)
O5—C9—C7	121.6 (4)	C13—C14—H14A	109.4
N3—C9—C7	116.3 (3)	C15—C14—H14A	109.4
N2—C7—C9	110.4 (3)	C13—C14—H14B	109.4
N2—C7—C8	109.9 (4)	C15—C14—H14B	109.4
C9—C7—C8	109.0 (4)	H14A—C14—H14B	108.0
N2—C7—H7	109.2	C14—C15—Br1	111.9 (4)
C9—C7—H7	109.2	C14—C15—H15A	109.2
C8—C7—H7	109.2	Br1—C15—H15A	109.2
C7—C8—H8A	109.5	C14—C15—H15B	109.2
C7—C8—H8B	109.5	Br1—C15—H15B	109.2
H8A—C8—H8B	109.5	H15A—C15—H15B	107.9
			
O3—S1—C1—C2	148.8 (4)	O4—S1—N2—C7	−50.8 (3)
O4—S1—C1—C2	21.1 (4)	C1—S1—N2—C7	62.9 (3)
N2—S1—C1—C2	−94.3 (4)	S1—N2—C7—C9	−95.6 (4)
O3—S1—C1—C6	−28.7 (4)	S1—N2—C7—C8	144.0 (4)
O4—S1—C1—C6	−156.4 (4)	O5—C9—C7—N2	−45.2 (5)
N2—S1—C1—C6	88.2 (4)	N3—C9—C7—N2	137.9 (4)
C6—C1—C2—C3	−1.3 (7)	O5—C9—C7—C8	75.7 (6)
S1—C1—C2—C3	−178.8 (4)	N3—C9—C7—C8	−101.3 (5)
C2—C1—C6—C5	−2.4 (7)	O5—C9—N3—C10	−2.8 (7)
S1—C1—C6—C5	175.0 (4)	C7—C9—N3—C10	174.1 (4)
C2—C1—C6—N1	178.3 (4)	C9—N3—C10—C12	−152.0 (4)
S1—C1—C6—N1	−4.3 (6)	C9—N3—C10—C11	87.0 (5)
C3—C4—C5—C6	−1.7 (8)	O6—C12—C10—N3	−52.6 (5)
C1—C6—C5—C4	3.9 (7)	N4—C12—C10—N3	130.4 (4)
N1—C6—C5—C4	−176.8 (5)	O6—C12—C10—C11	68.6 (5)
C1—C2—C3—C4	3.5 (8)	N4—C12—C10—C11	−108.4 (4)
C5—C4—C3—C2	−1.9 (9)	O6—C12—N4—C13	5.1 (7)
C1—C6—N1—O1	−67.9 (6)	C10—C12—N4—C13	−178.0 (4)
C5—C6—N1—O1	112.8 (5)	C12—N4—C13—C14	143.2 (5)
C1—C6—N1—O2	114.4 (5)	N4—C13—C14—C15	−174.7 (4)
C5—C6—N1—O2	−64.9 (6)	C13—C14—C15—Br1	177.8 (4)
O3—S1—N2—C7	179.3 (3)		

### Hydrogen-bond geometry (Å, °)

**Table:**

*D*—H···*A*	*D*—H	H···*A*	*D*···*A*	*D*—H···*A*
N2—H2A···O6^i^	0.86	2.07	2.884 (4)	158
N3—H3A···O6	0.86	2.54	2.829 (4)	100
N3—H3A···O5^ii^	0.86	2.07	2.899 (4)	162
N4—H4A···O4^i^	0.86	2.35	3.165 (5)	159
C2—H2···O4	0.93	2.49	2.867 (6)	104
C7—H7···O5^ii^	0.98	2.34	3.193 (5)	145
C10—H10···O4^i^	0.98	2.51	3.431 (5)	156
C13—H13A···O6	0.97	2.46	2.802 (6)	100
C13—H13B···O3^iii^	0.97	2.60	3.496 (6)	154
C11—H11A···Cg	3.39	0.96	3.922 (6)	117
C11—H11B···Cg^i^	3.27	0.96	3.857 (6)	121

Symmetry codes: (i) *x*−1/2, −*y*+1/2, −*z*+2; (ii) *x*+1/2, −*y*+1/2, −*z*+2; (iii) *x*, *y*−1, *z*.
